# Proteomics in Traditional Chinese Medicine with an Emphasis on Alzheimer's Disease

**DOI:** 10.1155/2015/393510

**Published:** 2015-10-18

**Authors:** Yanuar Alan Sulistio, Klaus Heese

**Affiliations:** Graduate School of Biomedical Science and Engineering, Hanyang University, 222 Wangsimni-ro, Seongdong-gu, Seoul 133-791, Republic of Korea

## Abstract

In recent years, there has been an increasing worldwide interest in traditional Chinese medicine (TCM). This increasing demand for TCM needs to be accompanied by a deeper understanding of the mechanisms of action of TCM-based therapy. However, TCM is often described as a concept of Chinese philosophy, which is incomprehensible for Western medical society, thereby creating a gap between TCM and Western medicine (WM). In order to meet this challenge, TCM research has applied proteomics technologies for exploring the mechanisms of action of TCM treatment. Proteomics enables TCM researchers to oversee various pathways that are affected by treatment, as well as the dynamics of their interactions with one another. This review discusses the utility of comparative proteomics to better understand how TCM treatment may be used as a complementary therapy for Alzheimer's disease (AD). Additionally, we review the data from comparative AD-related TCM proteomics studies and establish the relevance of the data with available AD hypotheses, most notably regarding the ubiquitin proteasome system (UPS).

## 1. Introduction

Traditional Chinese medicine (TCM) has promising potential as a complementary or alternative therapy for the treatment of neurodegenerative diseases (NDs). In recent years, TCM has become increasingly popular in Western countries [1]. The mechanisms of TCM on cellular levels, however, are poorly understood due to the complexity of the active components of TCM and the poor documentation available for mechanistic studies. TCM often utilizes several active ingredients, which may have either synergistic or antagonistic effects on cells. Accordingly, with the use of “omics” methods such as proteomics, the mechanisms of TCM treatment can be better explained and more fully understood [2, 3].

Alzheimer's disease (AD) is an ND that causes patients to exhibit high cognitive dysfunction, memory impairment, language deterioration, depression, and other debilitating conditions caused by the death of neurons in specific areas of the brain [4, 5]. Numerous TCM treatments have been reported to be effective therapies for AD [6–9]. The therapies, however, are still lacking sound scientific explanations. Comparative proteomics studies of TCM, which compare the relative quantity of proteomes between control and TCM-treated cells, may provide a holistic perspective on the mechanisms of active TCM phytochemicals [10, 11]. In this review, we focus on the proteomics approaches of existing TCM studies and on the relevance of these approaches in deciphering general TCM mechanisms for the treatment of NDs such as AD. Further, we emphasize AD as an example of how proteomics studies are able to explain the ways in which TCM treatments have positive effects at cellular and molecular levels.

## 2. TCM

TCM is a therapeutic system that has been practiced for more than 2,000 years, making it one of the oldest medical systems in the world [12, 13]. It is influenced by ancient Chinese philosophy and the idea that coherency between nature and human beings has a vital effect upon the health of people [12]. TCM is distinct from conventional Western medicine (WM) in many aspects. First of all, the primary difference is the fundamental theory of medicine [14]. Unlike WM wherein diseases are explained theoretically using biology and physiology, TCM etiologies of diseases are described by theories from ancient Chinese philosophy [13]. Secondly, TCM formulas are often mixtures of several herbs, in which each component may have several active ingredients which can interact with each other in any number of ways, including mutual accentuation, mutual enhancement, mutual counteraction, mutual suppression, mutual antagonism, and mutual incompatibility [15]. In contrast, WM often only consists of a single active compound. Next, the emergence of TCM and WM is greatly divergent. TCM was developed over a couple of millennia and is practiced naturally by a massive population in East Asia as a folk remedy, while WM has been driven by scientific researches [1]. Nevertheless, since the 1950s, TCM has experienced a trend toward modernization and increased acceptance in Western countries [16]. This phenomenon demands more TCM research to establish scientific mechanistic studies, safety assessments, and standardized manufacturing practices.

## 3. Proteomics and TCM

The biggest challenge for the modernization of TCM involves unraveling the complexities of TCM mechanisms with sound scientific principles [17]. To achieve this, TCM research has applied “omics” methods in order to elucidate the complex network of TCM mechanisms [2]. “Omics” methods, such as proteomics, offer advantages for understanding the disease in the bigger picture and can reveal the dynamic interactions between the active components of TCM formulas [2]. Consequently, proteomics has become a crucial tool for deciphering the intricate mechanisms of TCM. To achieve this goal of broader empirical understanding, three proteomics strategies are available for TCM researchers. These strategies are syndrome proteomics, screening proteomics, and comparative proteomics.

### 3.1. Syndrome Proteomics Strategy

The first proteomics strategy is syndrome proteomics, which is used for translating a syndrome, as it is understood in TCM terminology, to biological principles [18]. This can be achieved by way of proteomics analysis of the organs or bodily fluids related to defined TCM syndromes. For example, the work of Sun et al. uses a stress-induced Gan-stagnancy syndrome model involving rats and 2D protein electrophoresis (2DE) proteomics to determine differentially regulated proteins in blood and tissues [19].

### 3.2. Screening Proteomics Strategy

The second proteomics strategy is screening proteomics. This strategy intends to elucidate the mechanisms of medicinal herbs used in TCM by identifying the binding partners of the active ingredients. This strategy is carried out by immobilizing target phytochemicals with an immobilized matrix. Subsequently, the whole proteome of a targeted tissue or plasma is screened through the immobilized phytochemical. Proteins that have the ability to bind to the phytochemicals will be immobilized in the matrix, while noninteracting proteins will be washed away. The immobilized proteins are then eluted and identified, in some cases, by using tandem mass spectrophotometry (MS/MS). This strategy is adopted in research to identify the molecular targets of curcumin, for example [20].

### 3.3. Comparative Proteomics Strategy

The last strategy is comparative proteomics, otherwise known as differential proteomics, which quantitatively determines the relative or absolute amount of proteins in TCM-treated and control groups and determines the key proteins altered between the groups. This strategy is widely adopted in the field of TCM research for determining the molecular actions of a TCM treatment. To date, there are several comparative proteomics techniques available for TCM researchers [21, 22]. The most prominent proteomics technique used in TCM studies is 2DE [2, 21, 22]. In this method, the whole proteome of a cell is separated based on the isoelectric point of the proteins on the first dimension, followed by a standard protein separation according to molecular weight on the second dimension (see Supplementary Figure S1 in Supplementary Material available online at http://dx.doi.org/10.1155/2015/393510). Consecutively, the proteomics profiles of the control or diseased cells and the TCM-treated cells are compared to find distinct protein expressions. Some of the proteins from the TCM-treated cells may be overexpressed or underexpressed in comparison to proteins in the control group. The differentially regulated proteins are the proteins of interest, and these are subsequently excised from the gel. The proteins of interest are then subjected to protein digestion, chromatography purification (e.g., by high performance liquid chromatography (HPLC)), and mass spectrophotometry (MS) fingerprinting or MS/MS sequencing to identify and quantify the proteins. An additional step involving a Western blot may be added to verify the differentially expressed proteins by comparing blots of control and TCM-treated cell protein extracts, as well as immunocytochemistry.

In addition to 2DE, there are several other methods to quantitatively assess the proteomics profiles of cell extracts. Stable-isotope labeling by amino acids in cell cultures (SILAC)* in vitro* is a proteomics method, which uses “light” or “heavy” isotope labeled amino acids to label respective (i.e., control and TCM-treated) samples (Supplementary Figure S2) [23]. These amino acids are introduced to the cell culture media and are then incorporated with the cellular proteins during cell growth and proliferation. The protein extracts for each sample are combined and then digested by protease (e.g., trypsin) and purified. Consequently, during MS/MS analysis, the peaks will appear as a pair: one with a lower mass-to-charge ratio (*m*/*z*), or the “lighter” peak (i.e., the control), and the other with a higher *m*/*z*, or the “heavier” peak (i.e., the TCM-treated sample). The relative amount of the respective proteins can be determined by comparing the respective peaks of the control and TCM-treated groups. An example of SILAC proteomics in TCM research is the identification of protein targets for celastrol, a phytochemical derived from* Tripterygium wilfordii*, in lymphoblastoid cells [24].

Alternatively, proteomics quantification can be achieved with protein-tagging methods, such as isobaric tags for relative and absolute quantification (iTRAQ) or tandem mass tags (TMT) [22]. These methods label the proteins after cell extraction according to the chemical reactions between the tag reagents and N-terminus of the peptides or the *ε*-amino group of lysine residue. The tags are composed of a reporter region, a balancer region, and a reactive region. The total mass of the tags is identical; hence the tags are isobaric, which is achieved by the inverse relationship of the mass in the reporter and balancer regions. A general overview of both the iTRAQ and TMT methods is as follows: the proteins are first extracted from the cells/tissue; then the proteins are digested to generate shorter peptides, which are then reacted with the tagging chemicals (e.g., 114 Da-reporter tag for the control group and 117 Da-reporter tag for the TCM-treated group) (Supplementary Figure S3). The peptides from both the control and TCM-treated groups are then combined and are subsequently purified to remove excess detergents that may interfere with the MS analysis. Following this step, the peptide solution may also be fractionated for preventing overcrowding of the peaks. The prepared peptides then undergo MS/MS analysis. In the first MS spectra, peptides from the control and treatment groups will appear as identical peaks, because the tags are still intact. During collision induced disassociation (CID), however, the tags will be fragmented, leaving only reporter ions, which appear as distinct peaks according to *m*/*z*. During MS/MS analysis, the peptide sequence is identified from the spectra, while the relative amounts of peptides in the control and treatment groups are deduced from the reporter ion peaks. For further discussion of the iTRAQ and TMT methods, readers are directed to other reviews [25, 26].

The advancement of proteomics technologies has made it possible for research to opt for the label-free method [27]. In this method, control and treated groups are prepared and analyzed in parallel (Supplementary Figure S4). In label-dependent proteomics, samples from control and TCM-treated groups are combined at some point after protein extraction. In label-free proteomics, however, the sample control and TCM-treated groups are processed separately. The relative quantification of proteins is achieved by comparing the spectra of the respective samples. This method offers a simple and streamlined protocol in comparison to other methods. However, this method may have higher variability due to the discrete processing of separate groups. A systemic comparison of the proteomics methods described above has been done in a previous study [28]. This comparison may provide insight for TCM researchers to design optimal proteomics experiments.

## 4. Alzheimer's Disease

Alzheimer's disease (AD) is the most common form of dementia, in which patients suffer from loss of higher cognitive functioning, memory impairment, language deterioration, depression, and other debilitating conditions caused by the death of neurons of specific areas of the brain [4, 29]. This disease is named after a German neuropathologist, Alois Alzheimer, who first presented a patient case for Auguste Deter in 1906. AD is characterized by hallmark pathological markers, which are aggregates of amyloid plaques (APs) and neurotoxic neurofibrillary tangles (NFTs). APs are composed of aggregated 40 or 42 amino acid amyloid beta peptides (A*β*_40/42_), while NFTs are composed of aggregated hyperphosphorylated Tau proteins. These pathological markers become more pronounced as the disease progresses in the brain [30]. The first area of the brain to be impacted by AD is the transentorhinal region, followed by the hippocampus, amygdala, and frontal lobe areas [31, 32].

AD can be initiated by genetic or nongenetic causes. A small proportion of AD is caused by a genetic mutation of the A*β* precursor protein (APP), microtubule-associated protein Tau (MAPT), and the presenilins-1 and presenilins-2 (PS1 and PS2, resp.) genes [4]. The mutations in APP, PS1, or PS2 genes shift the production of the A*β* precursor protein toward the amyloidogenic pathway [33–35]. The genetic type of AD usually has an earlier onset; hence it is also known as early-onset AD. The other type of AD is caused by nongenetic factors, with onset at a later age. This type of AD is termed sporadic AD or late-onset AD [36].

There are several hypotheses about the pathogenesis of AD, leading to eventual neuronal death. One of the earliest hypotheses is the cholinergic hypothesis [37–39]. The cholinergic hypothesis is underlined by evidence of the loss of cholinergic neurons in the brain, which ultimately results in cognitive decline in AD patients [40]. According to this hypothesis, the proposed causes of neuronal death include reduced expression of acetylcholine receptors, decreased production of the acetylcholine neurotransmitters, and impaired axonal transport. This results in a failure to maintain synaptic connections between neurons, thus triggering neuronal death in AD patients.

The second hypothesis about the pathogenesis of AD is the amyloid cascade hypothesis, which is the most prominent AD hypothesis to date. This hypothesis reasons that accumulation of A*β* causes the formation of NFTs in neurons and eventually induces apoptosis [41, 42]. A*β* is a product of the APP protein when cleaved by *β*- and *γ*-secretases by a mechanism called regulated intramembrane proteolysis (RIP) [43, 44]. Cytotoxic A*β* may cause cell death by inducing oxidative stress, calcium imbalance, and mitochondrial damage or by disturbing energy production, inducing Tau protein phosphorylation, and/or impairing the protein degradation system [45–52].

The next hypothesis is the Tau protein hypothesis [53, 54]. Tau proteins are a component of microtubules and are imperative for maintaining microtubule architecture in the axons [55, 56]. However, Tau proteins are easily hyperphosphorylated, which results in loss-of-function and in turn causes the loss of attachment to the microtubule [53, 57]. Detached Tau proteins may interfere with the axon's ability to maintain axonal transport, eventually resulting in synaptic dysfunction and neuronal death [54, 58, 59]. Tau protein hyperphosphorylation can be reversed with the application of protein phosphatases such as protein phosphatase 2A (PP2A) or protein phosphatase 5 (PP5), which restores the ability of Tau proteins to stabilize microtubules [60, 61]. Conversely, in terms of AD, Tau protein hyperphosphorylation can be triggered by the reduced expression or activity of phosphatases [60, 62].

In recent years, a paradigm shift has occurred in AD research due to the failure of preexisting theories to provide a satisfactory explanation of AD pathophysiology and effective therapeutic strategies [63–66]. Researchers are now investigating the process of regulating misfolded proteins to gain alternative hypotheses for explaining AD and other NDs and ultimately for the development of new strategies for the treatment of AD. Misfolding-prone proteins are classified as intrinsically disordered proteins (IDPs), a group of proteins lacking defined tertiary structures, which are thus susceptible to aggregation [67]. Many representative proteins of NDs, such as A*β*, Tau proteins, *α*-synuclein, superoxide dismutase-1 (SOD-1), and TAR DNA-binding protein 43 (TDP-43), are included in this group [68–72]. In particular, this hypothesis emphasizes the importance of the ubiquitin proteasome system (UPS) and molecular chaperones for preventing the aggregation of IDPs [73, 74]. The UPS is responsible for degrading the majority of cellular proteins and maintaining protein homeostasis and is also able to degrade misfolded proteins [75, 76]. The ubiquitin proteasome system consists of the 26S proteasome as the proteolytic complex, ubiquitin ligases as the targeting mechanism, and ubiquitin recycling enzymes [76–78]. While the 26S proteasome is a protein complex comprised of a 20S proteasome catalytic core capped with a pair of 19S regulatory complexes, it has been numerously reported that inhibition of this proteasomal system leads to perturbed degradation of A*β* and hyperphosphorylated Tau protein, which eventually leads to an accumulation of these proteins and cytotoxicity [79]. The 20S proteasome core itself is composed of two *α*- and *β*- rings with seven subunits for each ring. Subunits *β*1, *β*2, and *β*5 are the active sites with caspase-like, trypsin-like, and chymotrypsin-like activities, respectively [80, 81]. It has been demonstrated that the 20S proteasome is able to degrade APP or at least the C-terminus of APP [82]. This degradation is inhibited by the addition of MG132, a proteasome inhibitor that targets the *β*5 subunit, and to a lesser extent also the *β*1 subunit of the 20S proteasome [83]. Therefore, it appears that the *β*5 subunit is the key subunit for the degradation of targeted proteins, including APP. In addition, other studies found that Tau protein may be degraded independent of the 20S proteasome chymotrypsin-like activity [84].

Besides, the regulatory subunits of the 20S proteasome also play a decisive role in the proteasome activity. This fact is demonstrated by a study that showed that an upregulation of PA28 subunits improves the 20S proteasome ability to degrade proteotoxic substrates [85].

In AD, the specific pathways of the UPS are impaired, due possibly to inhibition on the catalytic core of the UPS by A*β* and aggregated Tau proteins, or due to decreased expression of ubiquitin-conjugating enzymes [48, 86–93]. Indeed, one study shows that ubiquitin overexpressing neurons have better survivability after ischemic stress in rodent brains [94]. The relationships among IDPs, the UPS, and molecular chaperones are also reviewed in other studies [73, 92, 95].

## 5. AD in TCM

The long history of dementia in TCM is documented in the books* Jingyue Quanshu* (1624 A.D.) and* Bian Zheng Lu* (1690 A.D.) [12]. The philosophy of TCM asserts that the brain is an outgrowth of and is nourished by kidney essence. It further explains that kidney essence produces the body's marrow, including cerebral marrow, spinal cord marrow, and bone marrow. When kidney essence is deficient, the production of cerebral marrow is reduced, which leads to various symptoms, including dementia. Therefore, according to TCM, AD is a deficiency of kidney essence [12]. Accordingly, AD treatment is achieved by tonifying kidney essence [96]. A more comprehensive TCM-based explanation states that AD, as well as other dementias, is caused by a deficiency of the vital energy of the kidneys (*Shen*), marrow (*Sui*), heart (*Xin*), or spleen (*Pi*), together with a stagnation of blood (*Xie*) and/or phlegm (*Tan*) [6]. Not surprisingly, TCM also perceives AD as the malfunctioning of multiple organs, including the kidneys, liver, heart, and spleen, in addition to the consequent accumulation of toxins and blood stasis [6, 9]. Hence, from a TCM point of view, effective treatment of AD must reverse the symptoms, as in a study that uses* Bushenhuatanyizhi* (an herbal mixture) to treat AD by bolstering kidney essence, removing phlegm, and promoting mental health [7].

**Figure 1:**
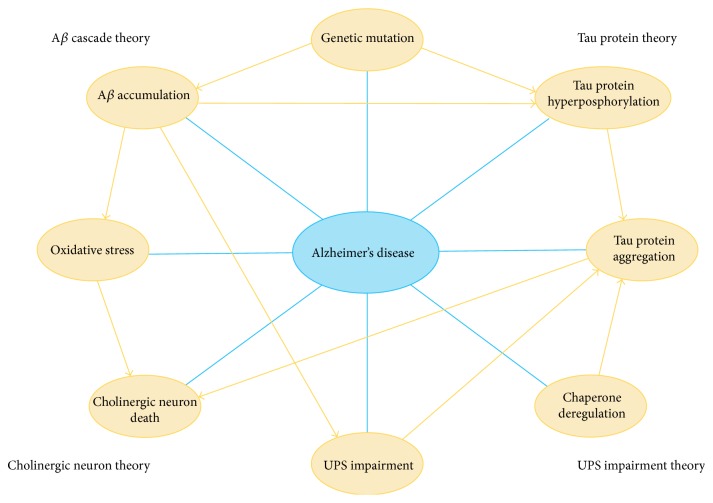
The multifaceted molecular pathology of AD. AD has been linked to many possible causes on genetic, molecular, and cellular levels. Each node in this figure represents a possible cause of AD. These causal events may work in concert and form an intricate cross-talking network, eventually resulting in neuronal death among patients.

Until recently, there has been no feasible treatment for AD. A great number of drugs have been developed based on existing AD theories, which have failed to be effective for treating or delaying AD [64, 65, 97, 98]. The drugs developed for AD thus far target only a single mechanism, while AD is often described as a multifaceted disease (meaning that the pathology cannot be described with only one theory) [64, 65, 97–101]. As described above, AD has several probable causes, which may interact with each other (Figure 1).

Under these circumstances, incorporating a TCM-based holistic perspective to the conventional AD treatment may be valuable.

In order to treat AD, TCM often uses a mixture of active ingredients with a diverse mechanistic action to target various molecular events. For instance,* Panax ginseng* extract has an array of active constituents that has been demonstrated* in vitro* to attack AD from different pathways. For example, ginsenoside Rg1 has been demonstrated to reduce apoptosis and to decrease the activity of *β*-secretase [102]. This activity is in synchrony with ginsenoside Rg3 that increases the expression of neprilysin, an A*β*-degrading enzyme, that further reduces the amyloid burden [103]. Moreover, another* Panax ginseng* component, the gintonin, has been reported to increase sAPP*α* release [104]. Further combination of several TCM herbs into a TCM mixture arguably enhances its potential. For example, an herbal mixture, composed of* Panax ginseng, Ginkgo biloba, and Crocus sativus* L., improves cognitive functions in double-transgenic APP (K670N/M671L)/PS1 (M146L) mice through reducing the amyloid burden [105]. The same study also concluded that the effects of this mixture are greater than the* Ginkgo biloba* extract alone. Therefore, a TCM approach could be more effective compared to a WM approach for treating a disease where the etiology is unknown, such as AD [13].

There is abundant literature describing TCM methods for treating AD. We provide an extensive list of AD-related TCM studies in Supplementary Table S1.

## 6. Comparative Proteomics of AD-TCM Research

Because TCM treatments often consist of several active ingredients that work in synergy to promote the activities of each component, conventional experiment methods, such as Western blotting or measuring single enzymatic activities, are often inadequate to study the effects of TCM treatment. To address this problem, TCM research has recently applied various “omics” techniques, such as proteomics. Comparative proteomics is able to shed new light on the possible mechanisms of several known natural ingredients used in TCM for treating AD and other cognitive dysfunctions. Thus far, comparative proteomics studies regarding AD-related TCM treatments are very limited.

### 6.1. *Huperzia serrata*

Huperzine A is sesquiterpene alkaloid compound that can be isolated from* Huperzia serrata* [106].* Huperzia serrata* is traditionally used in TCM as a treatment for fever, inflammation, blood disorders, myasthenia gravis, and schizophrenia [106, 107]. Huperzine A is also a promising drug candidate for the treatment of AD, with three meta-analysis studies reporting positive effects of the compound on patients with AD [108, 109].

In 1986, it was discovered that* Huperzia serrata*-derived Huperzine is a strong cholinesterase inhibitor [110, 111]. Cholinesterase is an enzyme that hydrolyzes the neurotransmitter acetylcholine into inactive metabolites [112]. Because it is suggested that AD is caused by acetylcholine deficiency, cholinesterase inhibitors, such as Huperzine A, are prescribed for AD treatment in China [107]. Emerging medicine, however, suggests that Huperzine A may also ameliorate AD via other mechanisms in addition to cholinesterase inhibition [107].

A proteomics study of Huperzine A on neuroblastoma N2a cells, which uses label-free liquid chromatography- (LC-) MS/MS, has discovered that Huperzine A has a neuroprotective effect on A*β*_1–42_ oligomers-induced toxicity [113]. The study compares the proteomics profiles of untreated N2a cells, A*β*-induced N2a, and A*β*-induced N2a treated with Huperzine A. The results reveal that 198 proteins are differentially regulated among the groups. The study finds that the Trp53 protein is downregulated by a fivefold measure in the group treated with Huperzine A. Additionally, among the 198 differentially regulated proteins, 15 proteins are found to interact directly with Trp53 in a protein-protein interaction network analysis.

The same study also reveals that proteins involved in the UPS are differentially regulated between the groups of patients with A*β*-induced N2a and A*β*-induced N2a treated with Huperzine A. The 26S proteasome core subunits, PSMB5 and PSMA5, and the 26S regulatory subunit, PSMD1, are found to be underexpressed upon A*β* induction and are rescued by the addition of Huperzine A. Other UPS components, such as E2 (UBE2K and CDC34) and E3 (UHRF1 and UBR4) ubiquitin ligases, are also regulated differentially. This argument is internally supported by the study's observation that Tau proteins are found to be lower in groups treated with Huperzine A. Tau proteins have been identified as the targets of the UPS. Thus, increased expression of proteins involved in the UPS ultimately leads to lower Tau protein accumulation. Another component of the UPS, UCHL3, which is a member of the ubiquitin C-terminal hydrolase (UCHL) family, is also shown to be upregulated in the Huperzine A-treated groups in the study. The implications of this protein are elucidated in Discussion of the present review.

An alternative possible mechanism of the Huperzine A-mediated amelioration of insults due to A*β* is the regulation of molecular chaperones and cochaperones, notably heat shock proteins 90 (HSP90) and HSP105, and the FK506 binding protein 8 (FKBP8, also known as FKBP38). HSP90, a pivotal chaperone for proper Tau protein folding and degradation, which has been shown to be downregulated in AD brains, has been found to be upregulated in the presence of Huperzine A [113, 114]. It has been suggested that HSP90 promotes the degradation of Tau proteins by recruiting the C-terminus of the HSP70-interacting protein (CHIP) E3 ligase and the promotion of Tau protein clearance [115]. The molecular cochaperone FKBP8 belongs to a group of proteins, so-called immunophilins, which are gaining attention for their role in AD pathogenesis [116]. The FKBP8 has been shown to promote apoptosis via interaction with presenilins [117]. In this study, FKBP8 is found to be inhibited by Huperzine A in A*β*-affected cells.

Furthermore, this study shows that Huperzine A may also support neuronal survival by upregulating PRDX3 [113], which is a member of the peroxiredoxin family of antioxidant enzymes that reduce hydrogen peroxide and alkyl hydroperoxides [118]. Upregulation of this protein may provide protection from oxidative insults induced by A*β*.

### 6.2. *Ginkgo biloba*

The earliest records of using* Ginkgo biloba* for the treatment of human diseases are documented in Li Tung-wan's* Shiwu Bencao* (Edible Herbal) and Wu-rui's Ri* Yong Ben Cao* (Herbal for Daily Usage) during the Yuan dynasty (1280–1368 A.D.), which chronicle the use of seeds for treatment of chronic bronchitis, asthma, enuresis, and tuberculosis [119]. Currently, extracts from* Ginkgo biloba* leaves are applied for the treatment of cognitive dysfunction, dementias, and AD [120].* Gingko biloba* is an excellent example of TCM that has been successfully commercialized and accepted as a food supplement in Western countries.* Ginkgo biloba* extract has been standardized as EGb 761 and is sold under several commercial names, including Tebonin, Tanakan, Rokan, Ginkoba, and Ginkgold [121]. Standardized EGb 761, comprising 24% flavonol glycosides and 6% terpenoids, is composed of bilobalide and the ginkgolides A, B, C, M, and J [122].

Clinical studies of* Ginkgo biloba* extract show different results. An electrophysiological study argues that chronic administration of* Ginkgo biloba* extract improves cognitive performance and increases steady-state visually evoked potential amplitudes in the frontal and occipital lobes of middle-aged people for the sake of solving working memory tasks, as recorded by scalp electrodes [123]. Some clinical trials also prove the effectiveness of EGb 761 over placebos [124]. In contrast, however, another study with an older median age of patients shows negative results [125]. Meta-analyses studies of* Ginkgo biloba* extract claim that* Ginkgo biloba* extract improves cognitive function in patients with dementia and AD [126, 127]. Many theories have been developed on how* Ginkgo biloba* extract acts on cognitive function [121]. The most widely accepted theory is that* Ginkgo biloba* extract-derived terpenoids act as antioxidants and radical scavengers [128–132].* Ginkgo biloba* extract also demonstrates neuroprotective ability against nitric oxide- (NO-) mediated toxicity by inhibiting protein kinase C (PKC) [133].

Vascular dementia (VD), the second leading cause of dementia, has some pathological overlap with AD and is suggested to have synergistic effects on AD [134–136]. In existing rodent models of VD, it has been observed that rodent brains demonstrate AD-characteristic pathogenesis following brain ischemia, such as the upregulation of APP [137], cleaving of APP into amyloid product [94, 138, 139], and Tau protein hyperphosphorylation [94, 140]. Due to this relationship, the data from VD studies can be carefully related to AD.

One proteomics study was carried out to determine the effects of* Ginkgo biloba* extract in rat brains after middle cerebral artery occlusion in a VD model [141]. Proteomics data from this study may shed light on protein regulation induced by a single dose of EGb 761. In the study, the author used 2DE and matrix-assisted laser desorption/ionization-time of flight (MALDI-TOF) MS for quantitative proteomics analysis. The proteomics analysis reveals that 23 proteins are differentially regulated (with a measure of change greater than 2.5) by EGb 761. The study emphasizes the deregulation of PRDX2 and PP2A subunit B (i.e., a protein with an official name of PPP2R4) following EGb 761 treatment upon occlusion of the middle cerebral artery in patients. The upregulation of PPP2R4 and PRDX2 may provide some insight into the beneficial effects of EGb 761 in the treatment of AD. PRDX2 is a member of the family of peroxiredoxin proteins, which has antioxidative functionality (see* Huperzia serrata*) [118].

The activator PPP2R4 works on the PP2A protein. With regard to AD, it has been theorized that A*β* may assert toxicity by activating cellular kinases that cause Tau protein hyperphosphorylation, eventually leading to aggregation [49]. PP2A is among the types of Tau protein-targeting phosphatases that prevent the aggregation of Tau proteins by dephosphorylating aggregation-prone hyperphosphorylated Tau proteins [61, 62]. It has been demonstrated that Tau protein dephosphorylation can restore the ability of Tau proteins to bind to microtubules [49, 54]. This finding is consistent with another study that reveals that EGb 761 is able to upregulate mRNA expression, as well as the activation of other phosphatases such as tyrosine/threonine phosphatase [142, 143].

Furthermore, this study also observes the downregulation of the HSP60 chaperone. The implication of HSP60 in AD is yet to be discovered. However, the modulation of HSPs is a common event in AD [144].

### 6.3. *Acanthopanax senticosus*

*Acanthopanax senticosus*, also known as* Eleutherococcus senticosus* or Siberian ginseng, has been used for treating various nervous and cardiovascular disorders [145]. This herb is reported to have positive effects on NDs such as amyotrophic lateral sclerosis (ALS), Parkinson's disease (PD), and AD [146–149].* Acanthopanax senticosus* extracts have been demonstrated to be neuroprotective both* in vitro* and* in vivo* [146, 149]. Additionally,* Acanthopanax senticosus* extract also prevents neuritic atrophy and potentially regenerates dystrophic neurites [147, 148]. Deeper investigation using proteomics methods may reveal the mechanisms of* Acanthopanax senticosus* extract in terms of AD treatment.

The work of Li et al. uses an iTRAQ quantitative proteomics method to decipher the effects of* Acanthopanax senticosus* extract on an* in vitro* PD model [150]. The authors prepare a crude extract derived from root and rhizomes of the herb using 80% ethanol, which is subsequently characterized by ultra-performance LC- (UPLC-) TOF/MS analysis. According to the results, the eleutheroside B and eleutheroside E content in* Acanthopanax senticosus* extract are 7.63 ± 0.34% (w/w) and 10.90 ± 0.22% (w/w), respectively.

In the study, the* Acanthopanax senticosus* extract is applied to SH-SY5Y neuroblastoma cells expressing A53T mutant *α*-synuclein. Although disturbed *α*-synuclein is more commonly observed in PD, a recent paradigm establishes a strong relationship between *α*-synuclein and Tau protein in AD as well [73, 151–153]. Accordingly, the results of this study may be carefully inferred to AD. The study finds that 84 proteins are differentially regulated between normal and A53T mutant *α*-synuclein-expressing cells. Among the 84 proteins, the expression of 16 proteins is reversed upon treatment with* Acanthopanax senticosus* extract.

The most significantly altered protein upon treatment with* Acanthopanax senticosus* extract is shown to be *α*-fetoprotein (AFP). AFP is upregulated in the SH-SY5Y overexpressing mutant *α*-synuclein, which is reversed upon treatment with the herbal extract. The authors conclude that the regulation of AFP is related to the apoptosis signal regulation. As it turns out, an additional insight can be deduced from this finding. AFP, together with *α*-synuclein and TDP-43, both of which are downregulated upon treatment with* Acanthopanax senticosus* extracts, are IDPs that are persistently found in NDs [68, 154]. Accumulation of IDPs is a pivotal indicator for the impaired clearance of misfolded proteins. The downregulation of IDPs upon treatment with the herbal extract suggests that* Acanthopanax senticosus* extracts have mechanisms for clearing misfolded proteins. Indeed, this is achieved by the drastic upregulation of PSMD7, a regulatory subunit of the 26S proteasome, in the* Acanthopanax senticosus* extract-treated cells. Overexpression of PSMD7 may recover the activity of the 26S proteasome, thus ensuring the proper clearance of misfolded proteins. In addition, the study shows that treatment with* Acanthopanax senticosus* extract causes an upregulation of USP5 (also known as UCHL5), which is another component of the UPS. The potential repercussions of the upregulation of UCHLs are elaborated on in Discussion of the present paper.

Another interesting finding from the study involves the upregulation of PPP2R5E, a regulatory subunit of PP2A. As described above (see* Ginkgo biloba*), PP2A prevents the aggregation of Tau proteins by dephosphorylating Tau proteins [61, 62].

Two members of HSP70 protein families, HSPA5 and HSPA9, are also found to be upregulated in* Acanthopanax senticosus* extract-treated cells. These HSPs are involved in protein processing in the endoplasmic reticulum and in protein folding [155]. However, the exact function of specific HSP molecules in AD is still controversial and is further assessed in our Discussion.

In addition, a comparative proteomics study for this herb has been carried out in an activated microglial cell line [156]. The authors of the study used bacterial LPS to stimulate inflammatory activation of the microglia. Activated microglia play an important role and may have interactive actions with the diseased neurons in AD pathogenesis [157, 158]. Therefore, assessing the effects of TCM extracts on microglia is critical to gain a better understanding of the effects of TCM on the AD brain. In the abovementioned study, proteome expression of cells treated with* Acanthopanax senticosus* extracts and a control group were compared using the 2DE method. The* Acanthopanax senticosus* extract significantly upregulated the expressions of PSMD13, FKBP4 (also known as FKBP52), and PRDX1. Although the specific functions of these proteins in microglia are not well characterized, this data suggests that there may be an interactive regulation of misfolded proteins between activated microglia and neurons during the progression of NDs [159–162]. Upregulation of the 26S proteasome subunit in microglia may help to degrade the misfolded proteins from the neighboring neurons, while the upregulation of PRDX proteins may help to battle oxidative insults [118]. FKBP4 has been demonstrated to inhibit Tau protein aggregation in neurons [163]. In sum, quantitative proteomics studies have shown that, in theory,* Acanthopanax senticosus* extracts have positive effects on AD patients by regulating protein clearance and Tau protein dephosphorylation in neurons and microglia.

### 6.4. *Gastrodia elata*

*Gastrodia elata*, commonly known as Tianma, is a member of the Orchidaceae family and is native to East Asian countries. Tianma has been used as traditional medicine for almost 2,000 years, as it was first described in an ancient Chinese medical text* Shennong Bencao Jing* (The Classic of Herbal Medicine) and also in* Bencao Gangmu* (Compendium of Materia Medica) [164, 165]. The tuber of Tianma has been used in TCM for centuries to treat dizziness, paralysis, and epilepsy [166]. Tianma is also used as an ingredient of* Tianma Gouteng Yin* and* Baizhu Tianma Tang* concoctions, which are prescribed for treating hypertension [167]. In addition to these health benefits, the use of Tianma has been suggested for treating cognitive dysfunction and NDs [168–170]. Tuber extracts from Tianma contain active ingredients of phenolic phytochemicals, where gastrodin and 4-hydroxybenzyl alcohol are the primary components [166].

Several publications indicate the potential benefits of Tianma extracts for the treatment of AD or VD [171–175]. A study using a rat model in which the rodents are injected with A*β*_25–35_ shows that long-term administration of crude Tianma water extract is partially effective for reversing A*β*-induced memory impairment [170]. Further, the study finds that Tianma extracts reduce the AP deposits and increase the expression and the activity of choline acetyltransferase. Similar results have been obtained by a different group of researchers using gastrodin instead of Tianma crude extracts [169]. Gastrodin also has been shown to be anti-inflammatory in the brains of mice and in cultured microglia cells. Gastrodin works by regulating the synthesis of proinflammatory cytokines [169, 176]. It is also reported that a water extract of Tianma has been known to modulate APP processing pathways, favoring the production of nonamyloidogenic products and enhanced cognitive functionality [171].

A comparative proteomics study using Tianma water extract has been carried out on neurons derived from human SH-SY5Y cells by using iTRAQ proteomics [177]. The study reveals that treatment with Tianma significantly alters the expression of 26S proteasome subunits, including PSMA1, PSMA2, PSMA3, PSMA4, PSMA5, PSMA6, PSMB7, PSMC3, PSMC5, PSMC6, PSMD1, PSMD2, PSMD3, PSMD8, PSMD11, PSMD12, PSMD13, and PSME3 [177]. As described, upregulation of 26S subunits may restore the protease abilities of the 26S proteasome [85]. Furthermore, the study demonstrates that TRIM28, a member of the E3 ligase protein family, is upregulated with treatment by Tianma. TRIM28 has been shown to target p53 for degradation; thus we assume that it has antiapoptotic effects [178]. Upregulation of these proteins may decrease the accumulation of misfolded proteins, thus preventing neurodegeneration. Similar to the active mechanism of* Acanthopanax senticosus* extracts, Tianma-treated cells have a threefold lower level of AFP in comparison to a control group of cells. This indicates that Tianma extracts may restore functionality in the UPS, which promotes the degradation of AFP. Two proteins from ubiquitin C-terminal hydrolase, UCHL3 and UCHL5, are also upregulated by treatment with Tianma [172]. The role of UCHLs in mediating TCM-treatment effects in AD is elaborated on in Discussion of the present review.

The same study also reveals that Tianma extracts may act on the regulation of molecular chaperones and cochaperones. Proteins from the HSPs families, such as HSP60, HSP70, HSP90, and HSP105, are found to be significantly upregulated by treatment with Tianma. Additionally, two FKBP immunophilins, FKBP3 and FKBP4, are also found to be upregulated by treatment with Tianma. We have discussed how HSP90 is involved in Tau protein degradation pathways (see* Huperzia serrata*), and it has been suggested that FKBP4 inhibits the aggregation of Tau proteins [163].

The effects of Tianma on the brain proteome have been elucidated* in vivo* in rats [171]. In accordance with the* in vitro* model, the investigation of brain proteomics reveals the involvement of HSP90 as a mediator protein in stimulation using Tianma. The brain proteomics of* in vivo* models, however, does not reveal the upregulation of 26S subunits, as found in* in vitro* models [171, 177]. This fact may be explained by the nature of the brain, in that the brain consists of a heterogeneous population of cells (i.e., mostly nonneuronal cells), which may mask the upregulation of neuron-enriched proteins [179, 180]. Additionally, tissue processing from* in vivo* studies involves treatments that are more elaborate, which may decrease the sensitivity of the proteomics assay. The study also reveals that PARK7 (also known as DJ-1), which is associated with autosomal recessive early-onset PD, is upregulated by Tianma [181]. It is suggested that the function of PARK7 is to protect cells from oxidative stress, and PARK7 may also act as a chaperone protein [182].

## 7. Discussion

There is a great deal of literature available to explain the mechanisms of TCM extracts in treating AD or improving general brain health. Meta-analyses and clinical trials of TCM-based treatment have shown the usefulness of TCM when used as prophylactic treatment for AD due to its improvement of cognitive function, improvement of daily life, and delay of cognitive decline in patients [124, 127, 183–186]. Thus far, available data often attributes the observed benefits of TCM limited to antioxidative action, free-radical scavenging, cholinesterase inhibition, or the antiapoptotic effects of TCM extracts (Supplementary Table S1). However, this is inadequate to fully explain the link between treatment and disease pathogenesis. The roles of antioxidants and cholinesterase inhibitors have been proven to be ineffective in treating or delaying AD [187–189], while apoptosis can be triggered by myriad direct or indirect causes, and therefore defining the apoptosis triggers (e.g., the inhibition of macroautophagy, DNA damage, accumulation of misfolded proteins, and other examples), rather than apoptosis itself, would be more beneficial for further mechanistic studies of TCM [190–192]. For these reasons, comparative proteomics studies of TCM are critical to discover further molecular mechanisms and to bridge the gap between TCM and WM.

**Figure 2:**
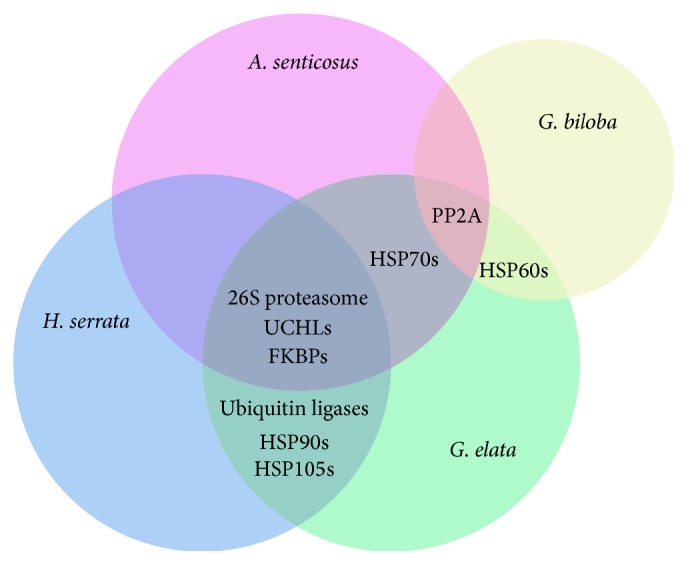
Diagram of the protein groups modulated by TCM treatment analyzed using proteomics methods. The UPS, consisting of the 26S proteasome, ubiquitin ligases, and ubiquitin hydrolases, is the main target of TCM treatments. Active TCM ingredients also target molecular chaperones and cochaperones such as HSPs and FKBPs. Additionally, TCM treatments modulate PP2A actions that regulate the dephosphorylation of Tau proteins.

The comparative proteomics data presented in this review reveals important mechanisms that are regulated upon treatment with TCM (Figure 2 and Table 1). The consistent involvement of the 26S proteasome and UPS are consistently observed in all experiments, with the exception of experiments related to* Ginkgo biloba*. Apart from this discrepancy, the data shows strong agreement on the upregulation of one or more 26S proteasome subunits in all cases. The ubiquitin ligases of the UPS are also found to be upregulated in the Huperzine A and Tianma studies, further suggesting that the UPS is a target of TCM phytochemicals. UPS pathways have been identified to degrade misfolded Tau proteins [115, 193–195]. Consequently, improving UPS activities by the overexpression of PA28*γ* (PSME3) recovers proteasome activities and bolsters cell survival of Huntington's disease-patient derived neurons [85]. Overexpression of PA28*α* (PSME1) also enhances the removal of misfolded and oxidized proteins and protects against oxidative stress in cardiomyocytes [196]. Thus, theoretically, the upregulation of UPS components by treatment with TCM is beneficial for AD patients.

Proteomics approaches are particularly beneficial for studying NDs, because NDs are characterized by the accumulation of protein aggregates, especially IDPs [70]. Through the use of proteomics, research is able to readily detect disordered proteins and to exploit them as internal indicators in support of the effects of TCM treatments. For example, in the Huperzine A study, Huperzine A upregulates the expression of UPS components, resulting in the more active proteasomal degradation of Tau proteins, thereby leading to lower observed levels of Tau proteins [113].

Additional findings from the proteomics data involve molecular chaperones and cochaperones. The most commonly occurring chaperones and cochaperones are HSP90, HSP70, HSP60s, HSP105s, and FKBPs. However, the role of HSPs in AD is still largely unknown. This ambiguity is caused by the ability of HSPs to bind promiscuously to a wide range of client proteins. Moreover, the actions of HSPs are often dictated by various regulator proteins, forming an HSP-client protein complex [144]. For instance, HSP70 and HSP90 have been known to form complexes with CHIP, an E3 ligase, to target Tau proteins for degradation by the UPS [115, 197–199]. One study claims that the forced induction of HSP70 and HSP90 decreases the aggregation of Tau proteins by promoting the binding of Tau proteins to microtubules [200]. Although HSP70 and HSP90 assist Tau protein degradation by recruiting CHIP, they also, to some extent, stabilize the binding of Tau proteins to microtubules, thus preventing the degradation of Tau proteins. In a seeming contradiction, it has been reported that the inhibition of HSP70 and HSP90 leads to the elimination of Tau protein aggregation [201–204]. Generally speaking, however, the expression of HSPs is inversely related to Tau protein aggregation, thus suggesting that the upregulation of HSPs by TCM is a favorable outcome [200].

Similar to HSPs, the role of FKBPs in AD is yet to be fully understood. A preliminary study argues that FKBP5 (FKBP51) promotes Tau protein accumulation, while FKBP4 (FKBP52) has a contrasting action by inhibiting the accumulation of Tau proteins [163]. It has also been suggested that FKBP4 protects against A*β* toxicity [205]. Accordingly, the upregulation of FKBP4 seems to be beneficial for treating AD, although there is no enough data to be conclusive about the benefits of other FKBPs. Smaller FKBPs, such as FKBP1A (FKBP12), have been found to coaggregate in NFTs [206]. It has been argued that the interaction between HSP and FKBP is important for Tau protein regulation [197]. Elucidating this link, however, is out of the scope of this review, and readers are directed to other reviews [197]. Because the function of FKBPs in AD is yet to be elucidated, the significance of FKBP modulation by TCM treatment is yet to be understood. However, it is important to recognize that the functions of individual members of FKBPs are very specific and cannot be generalized. For instance, FKBP4 and FKBP5 have almost identical protein domains, yet their actions on Tau protein regulation are antagonistic.

The proteomics data reviewed herein indicates that UCHL proteins are consistently modulated in most cases [113, 156, 177]. Although the potential involvement of this group of proteins in AD has been proposed, no strong conclusions have been reached [76, 91, 95, 207–218]. The UCHL protein group is important in maintaining availability of ubiquitin proteins for the UPS by recycling ubiquitin tags [211]. The best-known example of this group of enzymes is UCHL1 (also known as PARK5), which is known to serve a dual role in the UPS as a deubiquitinating enzyme in monomeric form or as an E3 ligase in dimeric form [215]. The loss-of-function of this particular protein has been demonstrated to impair the UPS and has been linked to the early-onset form of various progressive NDs, including PD [207, 219]. Furthermore, overexpression of UCHL1 reduces the production of A*β* by downregulating the protein level of *β*-secretase and APP [213, 214]. On the other hand, it has been reported that UCHL1, together with Parkin, promotes mitochondrial and synaptic failure by excessive activation of mitophagy in a truncated-Tau protein expressing cell line model [218]. UCHL3 is an important protein for functional working memory [212]. UCHL3 is also downregulated in an accelerated-senescence mouse model, suggesting that UCHL3 expression decreases with aging [216]. The implications of UCHL5 to proteasomal degradation are contradictory. It has been proposed that the expression of UCHL5 (UCH37) impedes proteasomal degradation by releasing polyubiquitin tags from the targeted proteins prior to introduction to the 26S proteasome [209]. One study reports that inhibition of UCHL5 with RNAi does not affect the proteolytic activity of the proteasome, but it does reduce the accumulation of polyubiquitinated proteins [217]. In contrast, other studies show that the inhibition of UCHL5 with the use of chemical inhibitors leads to accumulation of ubiquitin-positive aggregates [208, 210]. This may suggest that UCHL5 needs to be maintained at certain levels to be able to recycle ubiquitin without impeding proteasomal degradation. Ultimately, the impact of TCM treatment in the expression of UCHLs must be assessed further in order to elucidate the benefits of TCM treatment for NDs, including AD and PD.

Another key point from the studies reviewed herein is that the proteomics data from* in vitro* experiments provides more detailed proteomics profiles than the proteomics data from* in vivo* experiments.* In vitro* proteomics data, in general, covers more proteins and overestimates the expression levels of control and treatment groups in comparison to* in vivo* experiments. Such a case occurred in the* in vivo* experiments using* Ginkgo biloba* and Tianma, which, having fewer numbers of covered proteins, may have caused our analysis to misjudge the regulation of interesting proteins. This fact is possibly due to the heterogeneity of the cells in the brain used in* in vivo* studies, especially in the brains of rodents, which have approximately four times the number of nonneuronal cells compared to neurons [179]. This may cause neuron-enriched proteins, such as some of the component proteins of the UPS, to be underestimated by quantitative proteomics [180]. Moreover, this discrepancy may arise due to the process of tissue cell preparation, in which the process of protein extraction from* in vivo* tissue is lengthier and potentially harsher (e.g., tissue homogenization and liquid nitrogen grinding), thereby potentially reducing the yield of proteins relative to* in vitro* experiments. Nevertheless, future proteomics technologies may overcome such problems. Ultimately, it is important to realize that it is impossible to conclude that one model is more meaningful than another, particularly when studying a complex and systematic disease such as AD.

## 8. Conclusion

In conclusion, TCM has a long history of treating dementias, including AD. Western medicine may stand to benefit from the centuries worth of TCM knowledge about AD, provided scientific explanation is available to validate and/or fully elucidate the findings of TCM. Proteomics is an essential tool in providing important insights to explain the effects of TCM treatment on AD. Based on the proteomics data reviewed herein, we conclude that TCM is a useful complementary and alternative medicine (CAM) for treating or delaying the onset of AD in patients, particularly when utilized as prophylactic treatment in the form of food supplements before the onset of the disease. Proteomics data reveals that the potential mechanisms of action of TCM for the prevention of AD pathogenesis involve overexpressing antioxidant proteins, reducing the accumulation of misfolded proteins by improving the UPS, modulating the expression of protein chaperones and cochaperones (notably HSPs and FKBPs), and overexpressing Tau protein phosphatase.

## Supplementary Material

The supplementary material provides four schematic diagrams (Supplementary Figures S1-S4) with an overview about current state-of-the-art technologies in protemics that are currently widely applied in TCM such as 2DE proteomics, SILAC proteomics, iTRAQ or TMT proteomics, and label-free quantitative proteomics. In addition, a Supplementary Table S1 provides an overview of AD-related studies using TCM or TCM-derived traditional medicines.

## Figures and Tables

**Table 1:** AD-related protein families regulated by TCM as shown in various proteomics studies.

Protein function	Protein name	References
26S proteasome	20S proteasome subunits alpha	[113, 177]
	20S proteasome subunits beta	[113, 177]
	26S proteasome regulatory subunits ATPase	[177]
	26S proteasome regulatory subunits non-ATPase	[113, 150, 156, 177]
	26S proteasome activator subunit	[113]

Ubiquitin ligases	Ubiquitin-conjugating enzyme E2K	[113]
	Ubiquitin-conjugating enzyme E2-CDC34	[113]
	E3 ubiquitin-protein ligase UHRF1	[113]
	E3 ubiquitin-protein ligase UBR4	[113]
	Tripartite motif containing 28	[177]

Ubiquitin C-terminal hydrolase	Ubiquitin carboxyl-terminal esterase L3	[113, 177]
	Ubiquitin carboxyl-terminal hydrolase L5	[150, 177]

Chaperone sand cochaperones	Heat shock protein 60 kDa	[141, 177]
	Heat shock protein 70 kDa	[150, 177]
	Heat shock protein 90 kDa	[113, 171, 177]
	Heat shock protein 105 kDa	[113, 177]
	FK506 binding protein 3, 25 kDa	[177]
	FK506 binding protein 4, 59 kDa	[156]
	FK506 binding protein 8, 38 kDa	[113]

Antioxidant	Peroxiredoxin 1	[156]
	Peroxiredoxin 2	[141]
	Peroxiredoxin 3	[113]

Phosphatases	Protein phosphatase 2A activator, regulatory subunit	[141]
	Protein phosphatase 2, regulatory subunit B	[150]
