# Supplementary material for: Proteomics in Traditional Chinese Medicine with an Emphasis on Alzheimer's Disease
Source: Evid Based Complement Alternat Med. 2015 Oct 18;2015:393510. doi: 10.1155/2015/393510 (PMC4628675; doi:10.1155/2015/393510)
Supplement: Supplementary file 1 — The supplementary material provides four schematic diagrams (Supplementary Figures S1-S4) with an overview about current state-of-the-art technologies in protemics that are currently widely applied in TCM such as 2DE proteomics, SILAC proteomics, iTRAQ or TMT proteomics, and label-free quantitative proteomics. In addition, a Supplementary Table S1 provides an overview of AD-related studies using TCM or TCM-derived traditional medicines. [file 393510.f1.pdf]

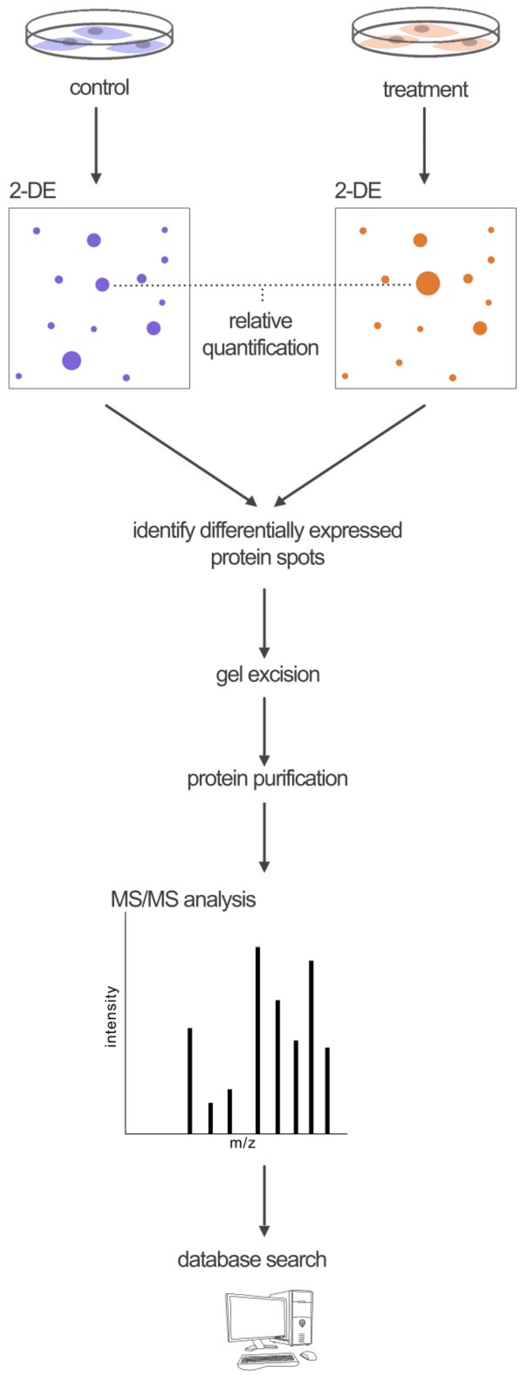

Supplementary Figure S1. Schematic diagram of 2DE proteomics. In this method, the proteins from treated cells and control proteins are extracted and separated using 2DE. The protein expression levels are compared by visualizing (e.g., by silver staining) and measuring the sizes of spots on the gel. The differentially expressed protein spots are then excised from the gel and purified (e.g., by HPLC) for MS/MS analysis.

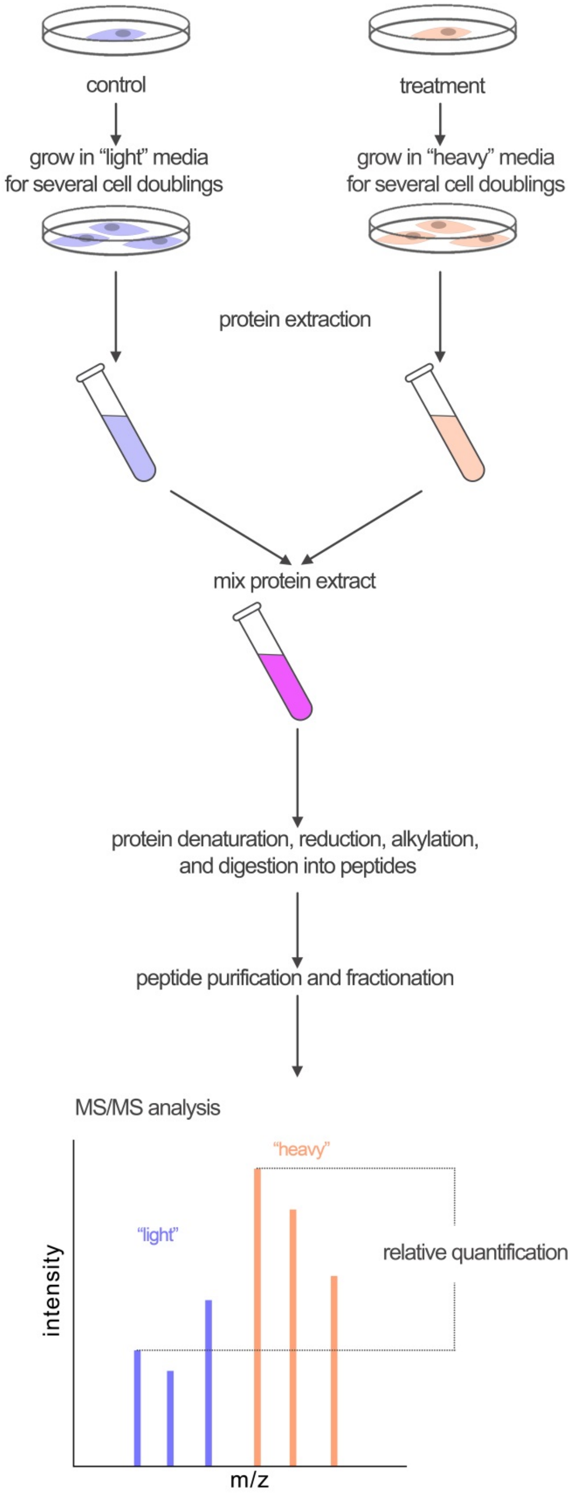

Supplementary Figure S2. Schematic diagram of SILAC proteomics. Cells from treatment and control groups are grown in media containing "heavy" or "light" amino acids for several cell doublings (i.e., at least five doublings) to incorporate the amino acids into the cellular proteins. Subsequently, the proteins from both groups are extracted and combined for purification (e.g., by HPLC), and are then subjected to MS/MS analysis. The MS/MS spectra will produce peaks with higher m/z for the "heavy" labeled proteins, depending on the number and mass of the "heavy" amino acids. Comparison of the two groups is done by comparing the intensity of the peaks of the "light" and "heavy" pairs.

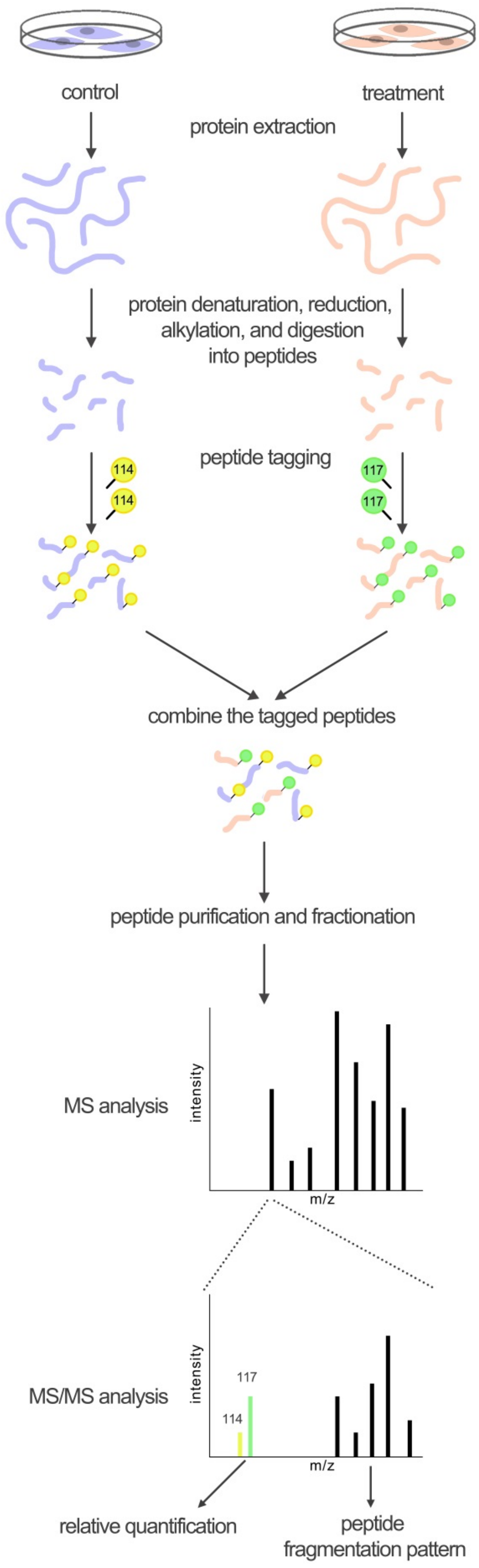

Supplementary Figure S3. Schematic diagram of iTRAQ or TMT proteomics. Cells from the treatment and control groups are extracted and digested into small peptides. These peptides then react with chemical labels (e.g., iTRAQ 114 and 117 tags for control and treatment groups, respectively). The tagged peptides are then combined and further purified (e.g., by HPLC) before undergoing MS/MS analysis. In the first MS spectrum, the peptides from both groups will produce a single peak. When the protein undergoes CID, however, the peptide will be fragmented, thereby releasing both the peptide and the balancer region from the tags. Ultimately, this process results in the production of peaks with discrete m/z measures according to the mass of the reporter region of the tag. Relative quantification of proteins is achieved by comparing the intensities of tags.

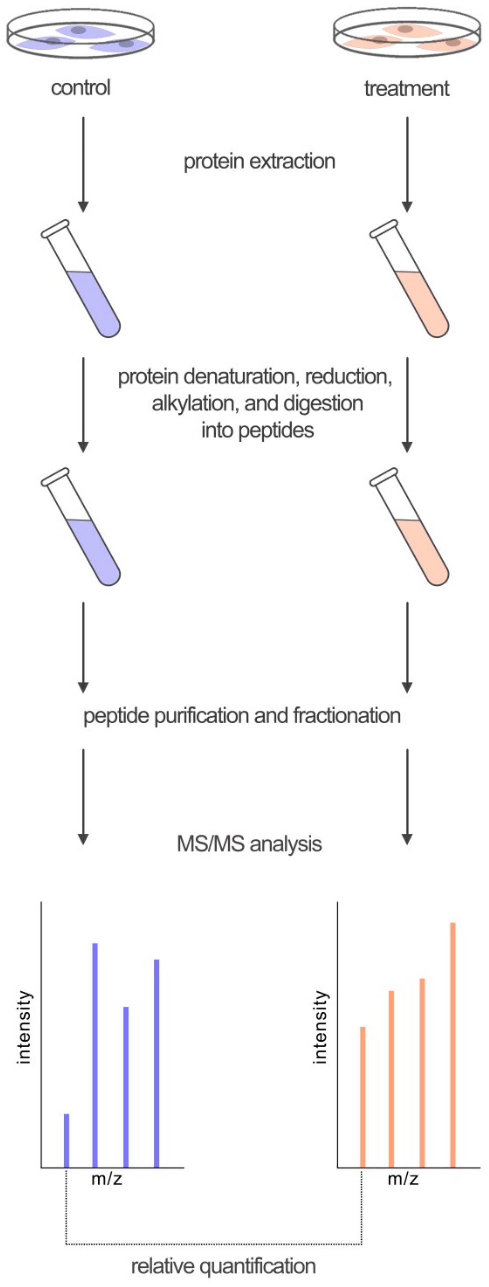

Supplementary Figure S4. Schematic diagram of label-free quantitative proteomics. In this method, protein extracts from the control and treatment groups are subjected to MS/MS analysis in parallel. The proteins from both groups are separately extracted, digested, purified (e.g., by HPLC), and analyzed using MS. Relative protein quantification is achieved by comparing the intensity of the identical peaks in respective samples.

Table S1. AD-related studies using TCM or TCM-derived traditional medicines.

**Title**	**Plant , TCM, or TCM-derived Material(s)**	**Testing Material(s)**	**Disease's model or assay**	**Effect of the TCM treatment**	**Ref**
Neuroprotective effect of garlic compounds in amyloid-beta peptide-induced apoptosis in vitro	*Allium sativum*	Aged garlic extract and S-allylcysteine	PC12 cells; Aβ25– 35	caspase-3 activation ↓, DNA fragmentation ↓, PARP cleavage ↓	[1]
S-allyl-L-cysteine selectively protects cultured rat hippocampal neurons from amyloid beta-protein- and tunicamycin-induced neuronal death	*Allium sativum*	S-allylcysteine	Primary rat hippocampal and cerebral granule neurons treated with Aβ and tunicamycin	ROS↓, caspase-3 activation ↓	[2]

Effect of aged garlic extract on caspase-3 activity, in vitro	*Allium sativum*	Aged garlic extract	Caspase-3 *in vitro* assay	caspase-3 activity↓	[3]
ESP-102, a standardized combined extract of Angelica gigas, Saururus chinensis and Schizandra chinensis, significantly improved scopolamine-induced memory impairment in mice	*Angelica gigas*, *Saururus chinensis*, and *Schizandra chinensis* in 8:1:1 ratio	ESP-102 (standarized extract)	Scopalamine treated mice, primary neuron treated with $A\beta_{25-35}$ and Glutamate	cognitive function↑, Aβ-induced toxicity↓	[4]
Differentiating effects of anisodamine on cognitive amelioration and peripheral muscarinic side effects induced by pilocarpine in mice	*Anisodus tanguticus*	Anisodamine	Mice	cognitive function↑	[5]

Antioxidant activity of Bacopa monniera in rat frontal cortex, striatum and hippocampus	*Bacopa monniera*	*Bacopa monniera* standardized extract	Rat	SOD , catalase , GPX activities↑	[6]
The green tea polyphenol (-)-epigallocatechin gallate attenuates beta-amyloid-induced neurotoxicity in cultured hippocampal neurons	*Camellia sinensis* (Green tea)	*Epigallocatechin gallate* (EGCG)	Primary neurons treated with Aβ	caspase-3 activation ↓, malondialdehyde↓	[7]
Effects of green tea polyphenol on cognitive and acetylcholinesterase activities	*Camellia sinensis* (Green tea)	Green tea-derived polyphenol	Scopolamine treated mice	cognitive function↑, acetylcholinesterase activity↓	[8]
Effect of *Centella asiatica* on cognition and oxidative stress in an intracerebroventricular streptozotocin model of Alzheimer's disease in rats	*Centella asiatica*	*Centella asiatica extract* (asiaticoside)	Straptozotocin treated rats	cognitive function↑, malondialdehyde activity ↓, glutathione, catalase activity ↑	[9]
Discovery of natural products from *Curcuma longa* that protect cells from beta-amyloid insult: a drug discovery effort against Alzheimer's disease	*Curcuma longa*	calebin-A, curcumin, demethoxycurcumin, bisdemethoxycurcumin, 1,7-	PC-12 cells treated with Aβ25– 35 or Aβ1–42	Aβ-induced toxicity↓	[10]

		bis(4-hydroxyphenyl)-1-heptene-3,5-dione			
Curcumin has potent anti-amyloidogenic effects for Alzheimer's beta-amyloid fibrils in vitro	*Curcuma longa*	Curcumin	Biochemical assay for Aβ1–40, Aβ1 – 42	Aβ aggregation ↓	[11]
Curcumin inhibits formation of amyloid β oligomers and fibrils, binds plaques, and reduces amyloid in vivo	*Curcuma longa*	Curcumin	Tg2576 mice	Aβ aggregation ↓	[12]
A water extract of *Curcuma longa* L. (Zingiberaceae) rescues PC12 cell death caused by pyrogallol or ypoxia/reoxygenation and attenuates hydrogen peroxide induced injury in PC12 cells	*Curcuma longa*	Water extract	PC12 cells treated with Pyrogallol or H2O2	antioxidant activities↑	[13]

The herbal medicine *Dipsacus asper Wall* extract reduces the cognitive deficits and overexpression of β-amyloid protein induced by aluminum exposure	*Dipsacus asper*	Crude extract	Aluminum chloride-induced AD rats	cognitive function↑, Aβ accumulation↓	[14]
Inhibitory effect of zeatin, isolated from *Fiatoua villosa*, on acetylcholinesterase activity from PC12 cells	*Fiatoua villosa*	Zeatin	Biochemical assay	acetylcholinesterase activity↓	[15]
Ginkgo biloba extract EGb 761 increases stress resistance and extends life span of Caenorhabditis elegans	*Ginkgo biloba*	EGb761	*C. elegans*	increase life span, ROS↓, apoptosis↓	[16]

Prevention of age-related spatial memory deficits in a transgenic mouse model of Alzheimer's disease by chronic Ginkgo biloba treatment	*Ginkgo biloba*	Commercial extract (similar to EGb761)	Tg2576 mice	cognitive function↑	[17]
Amyloid precursor protein metabolism is regulated toward alpha-secretase pathway by Ginkgo biloba extracts	*Ginkgo biloba*	EGb761	Rats	non-amyloidogenic APP processing↑	[18]
Effects of EGb 761 Ginkgo biloba extract on mitochondrial function and oxidative stress	*Ginkgo biloba*	EGb761	PC12 cells treated with Aβ	oxidative stress ↓, ROS↓, apoptosis↓ , Aβ-induced toxicity↓	[19]
Antiapoptotic properties of Ginkgo biloba extract EGb 761 in differentiated PC12 cells	*Ginkgo biloba*	EGb761	PC12 cells	caspase-3 activation ↓	[20]

The Ginkgo biloba extract Egb 761 rescues the PC12 neuronal cells from beta-amyloid-induced cell death by inhibiting the formation of beta-amyloid-derived diffusible neurotoxic ligands	*Ginkgo biloba*	EGb761	PC12 cells treated with Aβ	Aβ aggregation ↓	[21]
Reactive oxygen species-induced apoptosis in PC12 cells and protective effect of bilobalide	*Ginkgo biloba*	EGb761	PC12 cells treated with ROS	p53 and caspase-3 activation ↓	[22]
EGb 761 is a neuroprotective agent against beta-amyloid toxicity	*Ginkgo biloba*	EGb761	Rats primary hippocampal neurons treated with Aβ	Oxidative stress ↓, ROS↓, apoptosis↓, Aβ-induced toxicity↓.	[23]

The Ginkgo biloba extract (EGb 761) protects and rescues hippocampal cells against nitric oxide-induced toxicity: involvement of its flavonoid constituents and protein kinase C	*Ginkgo biloba*	EGb761	Rat primary mixed hippocampal cell cultures	NO-induced toxicity↓, PKC activity ↓	[24]
The Ginkgo biloba extract (EGb 761) protects hippocampal neurons against cell death induced by beta-amyloid	*Ginkgo biloba*	EGb761	Rats primary hippocampal neurons treated wih Aβ	ROS↓, apoptosis↓	[25]
Ginkgolide B inhibits the neurotoxicity of prions or amyloid-beta1-42	*Ginkgo biloba*	Ginkgolide B	SH-SY5Y treated with Aβ or prion proteins sPrP106	E2 prostalglandin production ↓, caspase-3 activation ↓, microglia activation ↓	[26]

Inhibition of amyloid-beta aggregation and caspase-3 activation by the Ginkgo biloba extract EGb761	*Ginkgo biloba*	EGb761	N2a cells treated with Aβ	Aβ aggregation ↓, caspase-3 activation ↓	[27]
Oxidative damage and protection by antioxidants in the frontal cortex of Alzheimer's disease is related to the apolipoprotein E genotype	*Ginkgo biloba*	EGb762	brain tissues from AD patients	lipid oxidation for patients with APO ε3/ ε3 and ε3/ ε4 allele↓ no effect on patients with ε4/ ε4 allele	[28]
Structure-activity studies with Ginkgo biloba extract constituents as receptor-gated chloride channel blockers and modulators	*Ginkgo biloba*	EGb 761	hippocampal and cerebellar neurons	blocks glycine-activated chloride channels	[29]

Self-assembly of Abeta(1– 42) into<br>globular neurotoxins	*Ginkgo biloba*	Ginkgo bilobaextract	PC12 cells treated with Aβ	Aβ aggregation ↓	[30]
Huperzine A enhances the level of<br>secretory amyloid precursor protein and protein kinase C-alpha in<br>intracerebroventricular beta-amyloid-(1– 40) infused rats and human<br>embryonic kidney 293 Swedish mutant cells	*Huperzia serrata*	Huperzine A	Sprague–Dawley rats; APPsw-expressing HEK293 cells	APP expression ↑, PKC activity ↑	[31]
Acetylcholinesterase complexed with<br>bivalent ligands related to huperzine A: experimental evidence for<br>species-dependent protein-ligand complementarity	*Huperzia serrata*	Huperzine A	*In vitro* cholinesterase inhibition assay	acetylcholinesterase activity↓	[32]
Huperzine A, a nootropic alkaloid, inhibits Nmethyl-d-aspartate-induced current in rat dissociated hippocampal neurons	*Huperzia serrata*	Huperzine A	Rats primary hippocampal neurons	NMDA-induced current ↓	[33]

Comparative effects of huperzine A, donepezil and rivastigmine on cortical acetylcholine level and acetylcholinesterase activity in rats	*Huperzia serrata*	Huperzine A	Rats	acetylcholinesterase activity↓	[34]
Indirubins inhibit glycogen synthase kinase-3 beta and CDK5/p25, two protein kinases involved in abnormal tau phosphorylation in Alzheimer's disease. A property common to most cyclindependent kinase inhibitors	*Indigo naturalis*	Indirubins	Insect Sf9 cells and tau phosphorylation in vitro; slices from adult mouse brain striatum	Tau protein phosphorylation↓, various kinases activities↓	[35]
Nicotine Enhances the Biosynthesis and Secretion of Transthyretin from the Choroid Plexus in Rats: Implications for β-Amyloid Formation	*Nicotiana tabaccum*	Nicotine	Rats	transthyretin (Aβ aggregation inhibitor) expression↑	[36]

Nicotine reduces Aβ in the brain and cerebral vessels of APPsw mice	*Nicotiana tabaccum*	Nicotine	APPsw-mutant mice	Aβ aggregation ↓	[37]
Nicotine increases the expression of high affinity nerve growth factor receptors in both in vitro and in vivo	*Nicotiana tabaccum*	Nicotine	Rats	TrkA expression ↑	[38]
Effect of combination of extracts of ginseng and *Ginkgo biloba* on acetylcholine in amyloid beta-protein-treated rats determined by an improved HPLC	*Panax ginseng* (Ginsenosides Rg1 and Re, 35%), *Ginkgo biloba* (Ginkgolides, 20%), and *Ginkgoflavones* (16%).	Naoweikang (Chinese traditional medicine)	Rats treated with Aβ	ACh level ↑	[39]
$A\beta_{25\text{-}35}$-induced memory impairment, axonal atrophy, and synaptic loss are ameliorated by M1, A metabolite of protopanaxadiol-type saponins	*Panax vietnamensis*	20-O-beta-D-glucopyranosyl-20(S)-protopanaxadiol and Ginsenoside Rb1	$A\beta_{25\text{–}35}$-injected mice and primary rat cortical neurons	Spatial memory↑, Neurite extension↑	[40]

Effect of kami-untan-to on the impairment of learning and memory induced by thiamine-deficient feeding in mice	*Pinellia ternate Breit* (3.0 g of tuber), *Poria cocos* (3.0 g of fungus), *Citrus unshiu* (3.0 g of peel), *Phyllostachys nigra* (3.0 g of stalk), *Zizyphus jujuba* (2.0 g of seed), *Scrophularia ningpoensis* (2.0 g of root), *Polygala tenuifolia* (2.0 g of root), *Panax ginseng* (2.0 g of root), *Rehmanii glutinosa* (2.0 g of root), *Zizyphus jujuba* (2.0 g of fruit), *Citrus aurantium* (2.0 g of immature fruit), *Glycyrrhiza glabra* (2.0 g of root), and *Zingiber officinable* (0.5 g of rhizome).	Kami-untan-to (Traditional Chinese and Japanese medicine)	Male ddY mice; Thiamine-deficient (TD) feeding	Choline acetyltransferase expression ↑	[41]

Tenuigenin treatment decreases secretion of the Alzheimer's disease amyloid beta-protein in cultured cells	*Polygala tenuifolia*	Tenuigenin	Neuroblastoma cells	β-secretase activity↓	[42]
Ptychopetalum olacoides, a traditional Amazonian ‘‘nerve tonic’’, possesses anticholinesterase activity	*Ptychopetalum olacoides*	*Ptychopetalum olacoides* ethanol extract	Frontal cortex, hippocampus, and striatal primary neurons of male Wistar rats (in vitro) and of male Swiss albino mice (ex vivo	acetylcholinesterase activity↓	[43]
Effects of Yukmijihwangtang derivatives (YMJd), a memory enhancing herbal extract, on the gene-expression profile in the rat hippocampus	*Rehmannia radix* (19.83%), *Discoreae radix* (20.05%), *Corni fructus* (41.64%), *Hoelen* (1.11%), *Mountain cortex radicis* (21.45%), and *Alismatis radix* (20.92%).	Yukmijihwang-tang (Chinese herbal medicine)	Rats	transthyretin, PEP-19 expression↑, apoptosis↓	[44]

Rhizoma acori graminei and its active principles protect PC-12 cells from the toxic effect of amyloid-beta peptide	*Acori graminei*	Eugenol and h-asarone	PC12 cells; Aβ1 – 40	Aβ-induced Ca^{2+} uptake↓	[45]
Protection against 1-methyl-4-phenylpyridinium ion (MPP+)-induced apoptosis by water extract of ginseng (*Panax ginseng* C.A. Meyer) in SH-SY5Y cells	*Panax ginseng*	Water extract of *Panax ginseng*	SH-SY5Y cells	ROS↓, apoptosis↓	[46]
Panax ginseng enhances cognitive performance in Alzheimer disease	*Panax ginseng*	*Panax ginseng* powder	AD patients	cognitive function ↑	[47]

An open-label trial of Korean red ginseng as an adjuvant treatment for cognitive impairment in patients with Alzheimer's disease.	*Panax ginseng*	Korean red ginseng (KRG)	AD patients	cognitive function↑	[48]
Long-term ginsenoside consumption prevents memory loss in aged SAMP8 mice by decreasing oxidative stress and upregulating the plasticity-related proteins in hippocampus	*Panax ginseng*	Crude ginsensodide	senescence-accelerated mouse	Aβ↓, anti-oxidant enzymes expression↑, neuronal plasticity-related proteins↑	[49]

The neuroprotective effects of ginsenosides on calcineurin activity and tau phosphorylation in SY5Y cells	*Panax ginseng*	Ginsenoside extracts from stems and leaves of *Panax ginseng*	SH-SY5Y cells	tau phosphorylation↓, anti-oxidant enzymes↑	[50]
Protective effect of ginsenoside Rb1 on beta-amyloid protein(1-42)-induced neurotoxicity in cortical neurons.	*Panax ginseng*	Ginsenoside Rb1	primary cortical neurons treated with Aβ	LDH, malonyldialdehyde, and SOD activity↓	[51]
Ginsenoside Rb1 protects PC12 cells against β-amyloid-induced cell injury	*Panax ginseng*	Ginsenoside Rb1	PC12 cells treated with Aβ	ROS↓, lipid peroxidation ↓, Bcl-2/Bax ratio↑	[52]

Involvement of calpain and p25 of CDK5 pathway in ginsenoside Rb1's attenuation of beta-amyloid peptide25-35-induced tau hyperphosphorylation in cortical neurons	*Panax ginseng*	Ginsenoside Rb1	Primary rat cortical neurons	p25 (CDK5 activator)↓, calpain activity↓, intracellular Ca^{2+}↓	[53]
Anti-neuroinfl ammation effect of ginsenoside Rbl in a rat model of Alzheimer disease.	*Panax ginseng*	Ginsenoside Rb1	Rat injected with $A\beta_{1-42}$	cognitive function↓, neuroinflammation↓	[54]
Ginsenoside-Rg1 inhibits cell apoptosis induced by beta amyloid	*Panax ginseng*	Ginsenoside Rg1	PS1M146L/APP751 mutant CHO cells	$A\beta_{42}$↓, caspase-3 activation↓	[55]

Ginsenoside Rg1 protects against hydrogen peroxide-induced cell death in PC12 cells via inhibiting NF-κB activation	*Panax ginseng*	Ginsenoside Rg1	PC12 cells treated with H_2O_2	NF-κB ↓, Akt and ERK1/2 ↑	[56]
Ginsenoside Rg1 attenuates amyloid-beta content, regulates PKA/CREB activity, and improves cognitive performance in SAMP8 mice	*Panax ginseng*	Ginsenoside Rg1	senescence-accelerated mouse	Aβ↓, PKA activity↓, pCREB↑, BDNF↑	[57]
Reductions in levels of the Alzheimer's amyloid beta peptide after oral administration of ginsenosides	*Panax ginseng*	Ginsenoside Rg1, Rg3, and Re	CHO cell line, Tg2576 mice	Aβ↓ (*in vitro* and in mouse brain)	[58]

Ginsenoside Rg1 inhibits betasecretase activity in vitro and protects against Abeta-induced cytotoxicity in PC12 cells	*Panax ginseng*	Ginsenoside Rg1	PC12 cells induced with Aβ	β-secretase activity↓, apoptosis↓	[59]
Ginsenoside Rg3 promotes beta-amyloid peptide degradation by enhancing gene expression of neprilysin	*Panax ginseng*	Ginsengoside Rg3	APP_{sw} SK-N-SH cells	neprylisin (Aβ-degrading enzyme) expression↑, Aβ↓	[60]
Gintonin, a ginseng-derived lysophosphatidic acid receptor ligand, attenuates Alzheimer's disease-related neuropathies: involvement of non-amyloidogenic processing	*Panax ginseng*	Gintonin	wt or mutant APP-expressing SH-SY5Y cells, various AD-phenotype mice	sAPPα release↑, $Aβ_{42}$↓, $Aβ_{40}$-mediated toxicity↓, AP formation↓ (*in vivo)*, cognitive function↑ (*in vivo*)	[61]

Protective effect of resveratrol on beta-amyloid induced oxidative PC12 cell death	*Vitis vinifera*	Resveratrol	PC12 cells treated with Aβ	ROS↓, apoptosis↓	[62]

References

1. Q. Peng, A.R. Buz'Zard, and B.H. Lau. "Neuroprotective effect of garlic compounds in amyloid-beta peptide-induced apoptosis in vitro," *Med Sci Monit, vol.* 8, no. 8, pp. BR328-337, 2002.
2. Y. Kosuge, Y. Koen, K. Ishige, et al. "S-allyl-L-cysteine selectively protects cultured rat hippocampal neurons from amyloid beta-protein- and tunicamycin-induced neuronal death," *Neuroscience, vol.* 122, no. 4, pp. 885-895, 2003.
3. R. Jackson, B. McNeil, C. Taylor, G. Holl, D. Ruff, and E.T. Gwebu. "Effect of aged garlic extract on caspase-3 activity, in vitro," *Nutr Neurosci, vol.* 5, no. 4, pp. 287-290, 2002.
4. S.Y. Kang, K.Y. Lee, K.A. Koo, et al. "ESP-102, a standardized combined extract of Angelica gigas, Saururus chinensis and Schizandra chinensis, significantly improved scopolamine-induced memory impairment in mice," *Life Sci, vol.* 76, no. 15, pp. 1691-1705, 2005.
5. H. Wang, Y. Lu, and H.Z. Chen. "Differentiating effects of anisodamine on cognitive amelioration and peripheral muscarinic side effects induced by pilocarpine in mice," *Neurosci Lett, vol.* 344, no. 3, pp. 173-176, 2003.
6. S.K. Bhattacharya, A. Bhattacharya, A. Kumar, and S. Ghosal. "Antioxidant activity of Bacopa monniera in rat frontal cortex, striatum and hippocampus," *Phytother Res, vol.* 14, no. 3, pp. 174-179, 2000.
7. Y.T. Choi, C.H. Jung, S.R. Lee, et al. "The green tea polyphenol (-)-epigallocatechin gallate attenuates beta-amyloid-induced neurotoxicity in cultured hippocampal neurons," *Life Sci, vol.* 70, no. 5, pp. 603-614, 2001.
8. H.K. Kim, M. Kim, S. Kim, M. Kim, and J.H. Chung. "Effects of green tea polyphenol on cognitive and acetylcholinesterase activities," *Biosci Biotechnol Biochem, vol.* 68, no. 9, pp. 1977-1979, 2004.
9. M.H. Veerendra Kumar and Y.K. Gupta. "Effect of Centella asiatica on cognition and oxidative stress in an intracerebroventricular streptozotocin model of Alzheimer's disease in rats," *Clin Exp Pharmacol Physiol, vol.* 30, no. 5-6, pp. 336-342, 2003.
10. S.Y. Park and D.S. Kim. "Discovery of natural products from Curcuma longa that protect cells from beta-amyloid insult: a drug discovery effort against Alzheimer's disease," *J Nat Prod, vol.* 65, no. 9, pp. 1227-1231, 2002.
11. K. Ono, K. Hasegawa, H. Naiki, and M. Yamada. "Curcumin has potent anti-amyloidogenic effects for Alzheimer's beta-amyloid fibrils in vitro," *J Neurosci Res, vol.* 75, no. 6, pp. 742-750, 2004.
12. F. Yang, G.P. Lim, A.N. Begum, et al. "Curcumin inhibits formation of amyloid beta oligomers and fibrils, binds plaques, and reduces amyloid in vivo," *J Biol Chem, vol.* 280, no. 7, pp. 5892-5901, 2005.
13. B.S. Koo, W.C. Lee, K.H. Chung, J.H. Ko, and C.H. Kim. "A water extract of Curcuma longa L. (Zingiberaceae) rescues PC12 cell death caused by pyrogallol or hypoxia/reoxygenation and attenuates hydrogen peroxide induced injury in PC12 cells," *Life Sci, vol.* 75, no. 19, pp. 2363-2375, 2004.

14. Z.J. Zhang, Y.H. Qian, H.T. Hu, J. Yang, and G.D. Yang. "The herbal medicine Dipsacus asper wall extract reduces the cognitive deficits and overexpression of beta-amyloid protein induced by aluminum exposure," *Life Sci, vol.* 73, no. 19, pp. 2443-2454, 2003.
15. H.J. Heo, S.C. Hong, H.Y. Cho, et al. "Inhibitory effect of zeatin, isolated from Fiatoua villosa, on acetylcholinesterase activity from PC12 cells," *Mol Cells, vol.* 13, no. 1, pp. 113-117, 2002.
16. Z. Wu, J.V. Smith, V. Paramasivam, et al. "Ginkgo biloba extract EGb 761 increases stress resistance and extends life span of Caenorhabditis elegans," *Cell Mol Biol (Noisy-le-grand), vol.* 48, no. 6, pp. 725-731, 2002.
17. R.W. Stackman, F. Eckenstein, B. Frei, D. Kulhanek, J. Nowlin, and J.F. Quinn. "Prevention of age-related spatial memory deficits in a transgenic mouse model of Alzheimer's disease by chronic Ginkgo biloba treatment," *Exp Neurol, vol.* 184, no. 1, pp. 510-520, 2003.
18. F. Colciaghi, B. Borroni, M. Zimmermann, et al. "Amyloid precursor protein metabolism is regulated toward alpha-secretase pathway by Ginkgo biloba extracts," *Neurobiol Dis, vol.* 16, no. 2, pp. 454-460, 2004.
19. A. Eckert, U. Keil, S. Kressmann, et al. "Effects of EGb 761 Ginkgo biloba extract on mitochondrial function and oxidative stress," *Pharmacopsychiatry, vol.* 36 Suppl 1, pp. S15-23, 2003.
20. J.V. Smith, A.J. Burdick, P. Golik, I. Khan, D. Wallace, and Y. Luo. "Anti-apoptotic properties of Ginkgo biloba extract EGb 761 in differentiated PC12 cells," *Cell Mol Biol, vol.* 48, no. 6, pp. 699-707, 2002.
21. Z. Yao, K. Drieu, and V. Papadopoulos. "The Ginkgo biloba extract EGb 761 rescues the PC12 neuronal cells from beta-amyloid-induced cell death by inhibiting the formation of beta-amyloid-derived diffusible neurotoxic ligands," *Brain Res, vol.* 889, no. 1-2, pp. 181-190, 2001.
22. L.J. Zhou and X.Z. Zhu. "Reactive oxygen species-induced apoptosis in PC12 cells and protective effect of bilobalide," *J Pharmacol Exp Ther, vol.* 293, no. 3, pp. 982-988, 2000.
23. S. Bastianetto and R. Quirion. "EGb 761 is a neuroprotective agent against beta-amyloid toxicity," *Cell Mol Biol (Noisy-le-grand), vol.* 48, no. 6, pp. 693-697, 2002.
24. S. Bastianetto, W.H. Zheng, and R. Quirion. "The Ginkgo biloba extract (EGb 761) protects and rescues hippocampal cells against nitric oxide-induced toxicity: involvement of its flavonoid constituents and protein kinase C," *J Neurochem, vol.* 74, no. 6, pp. 2268-2277, 2000.
25. S. Bastianetto, C. Ramassamy, S. Dore, Y. Christen, J. Poirier, and R. Quirion. "The Ginkgo biloba extract (EGb 761) protects hippocampal neurons against cell death induced by beta-amyloid," *Eur J Neurosci, vol.* 12, no. 6, pp. 1882-1890, 2000.
26. C. Bate, M. Salmona, and A. Williams. "Ginkgolide B inhibits the neurotoxicity of prions or amyloid-beta1-42," *J Neuroinflammation, vol.* 1, no. 1, p. 4, 2004.
27. Y. Luo, J.V. Smith, V. Paramasivam, et al. "Inhibition of amyloid-beta aggregation and caspase-3 activation by the Ginkgo biloba extract EGb761," *Proc Natl Acad Sci U S A, vol.* 99, no. 19, pp. 12197-12202, 2002.

28. C. Ramassamy, D. Averill, U. Beffert, et al. "Oxidative damage and protection by antioxidants in the frontal cortex of Alzheimer's disease is related to the apolipoprotein E genotype," *Free Radic Biol Med, vol.* 27, no. 5-6, pp. 544-553, 1999.
29. S.S. Chatterjee, E.L. Kondratskaya, and O.A. Krishtal. "Structure-activity studies with Ginkgo biloba extract constituents as receptor-gated chloride channel blockers and modulators," *Pharmacopsychiatry, vol.* 36 Suppl 1, pp. S68-77, 2003.
30. B.A. Chromy, R.J. Nowak, M.P. Lambert, et al. "Self-assembly of Abeta(1-42) into globular neurotoxins," *Biochemistry, vol.* 42, no. 44, pp. 12749-12760, 2003.
31. H.Y. Zhang, H. Yan, and X.C. Tang. "Huperzine A enhances the level of secretory amyloid precursor protein and protein kinase C-alpha in intracerebroventricular beta-amyloid-(1-40) infused rats and human embryonic kidney 293 Swedish mutant cells," *Neurosci Lett, vol.* 360, no. 1-2, pp. 21-24, 2004.
32. D.M. Wong, H.M. Greenblatt, H. Dvir, et al. "Acetylcholinesterase complexed with bivalent ligands related to huperzine a: experimental evidence for species-dependent protein-ligand complementarity," *J Am Chem Soc, vol.* 125, no. 2, pp. 363-373, 2003.
33. J.M. Zhang and G.Y. Hu. "Huperzine A, a nootropic alkaloid, inhibits N-methyl-D-aspartate-induced current in rat dissociated hippocampal neurons," *Neuroscience, vol.* 105, no. 3, pp. 663-669, 2001.
34. Y.Q. Liang and X.C. Tang. "Comparative effects of huperzine A, donepezil and rivastigmine on cortical acetylcholine level and acetylcholinesterase activity in rats," *Neurosci Lett, vol.* 361, no. 1-3, pp. 56-59, 2004.
35. S. Leclerc, M. Garnier, R. Hoessel, et al. "Indirubins inhibit glycogen synthase kinase-3 beta and CDK5/p25, two protein kinases involved in abnormal tau phosphorylation in Alzheimer's disease. A property common to most cyclin-dependent kinase inhibitors?," *J Biol Chem, vol.* 276, no. 1, pp. 251-260, 2001.
36. M.D. Li, J.K. Kane, S.G. Matta, W.S. Blaner, and B.M. Sharp. "Nicotine enhances the biosynthesis and secretion of transthyretin from the choroid plexus in rats: implications for beta-amyloid formation," *J Neurosci, vol.* 20, no. 4, pp. 1318-1323, 2000.
37. E. Hellstrom-Lindahl, J. Court, J. Keverne, et al. "Nicotine reduces A beta in the brain and cerebral vessels of APPsw mice," *Eur J Neurosci, vol.* 19, no. 10, pp. 2703-2710, 2004.
38. R.R. Jonnala, A.V. Terry, Jr., and J.J. Buccafusco. "Nicotine increases the expression of high affinity nerve growth factor receptors in both in vitro and in vivo," *Life Sci, vol.* 70, no. 13, pp. 1543-1554, 2002.
39. J.X. Liu, W.H. Cong, L. Xu, and J.N. Wang. "Effect of combination of extracts of ginseng and ginkgo biloba on acetylcholine in amyloid beta-protein-treated rats determined by an improved HPLC," *Acta Pharmacol Sin, vol.* 25, no. 9, pp. 1118-1123, 2004.
40. C. Tohda, N. Matsumoto, K. Zou, M.R. Meselhy, and K. Komatsu. "Abeta(25-35)-induced memory impairment, axonal atrophy, and synaptic loss are ameliorated by M1, A metabolite of protopanaxadiol-type saponins," *Neuropsychopharmacology, vol.* 29, no. 5, pp. 860-868, 2004.
41. O. Nakagawasai, F. Yamadera, K. Iwasaki, et al. "Effect of kami-untan-to on the impairment of learning and memory induced by thiamine-deficient feeding in mice," *Neuroscience, vol.* 125, no. 1, pp. 233-241, 2004.
42. H. Jia, Y. Jiang, Y. Ruan, et al. "Tenuigenin treatment decreases secretion of the Alzheimer's disease amyloid beta-protein in cultured cells," *Neurosci Lett, vol.* 367, no. 1, pp. 123-128, 2004.

43. I.R. Siqueira, C. Fochesatto, A.L. da Silva, et al. "Ptychopetalum olacoides, a traditional Amazonian "nerve tonic", possesses anticholinesterase activity," *Pharmacol Biochem Behav, vol.* 75, no. 3, pp. 645-650, 2003.
44. S. Rho, M. Kang, B. Choi, et al. "Effects of Yukmijihwang-tang derivatives (YMJd), a memory enhancing herbal extract, on the gene-expression profile in the rat hippocampus," *Biol Pharm Bull, vol.* 28, no. 1, pp. 87-93, 2005.
45. Y. Irie and W.M. Keung. "Rhizoma acori graminei and its active principles protect PC-12 cells from the toxic effect of amyloid-beta peptide," *Brain Res, vol.* 963, no. 1-2, pp. 282-289, 2003.
46. S. Hu, R. Han, S. Mak, and Y. Han. "Protection against 1-methyl-4-phenylpyridinium ion (MPP+)-induced apoptosis by water extract of ginseng (Panax ginseng C.A. Meyer) in SH-SY5Y cells," *J Ethnopharmacol, vol.* 135, no. 1, pp. 34-42, 2011.
47. S.T. Lee, K. Chu, J.Y. Sim, J.H. Heo, and M. Kim. "Panax ginseng enhances cognitive performance in Alzheimer disease," *Alzheimer Dis Assoc Disord, vol.* 22, no. 3, pp. 222-226, 2008.
48. J.H. Heo, S.T. Lee, K. Chu, et al. "An open-label trial of Korean red ginseng as an adjuvant treatment for cognitive impairment in patients with Alzheimer's disease," *Eur J Neurol, vol.* 15, no. 8, pp. 865-868, 2008.
49. H. Zhao, Q. Li, Z. Zhang, X. Pei, J. Wang, and Y. Li. "Long-term ginsenoside consumption prevents memory loss in aged SAMP8 mice by decreasing oxidative stress and up-regulating the plasticity-related proteins in hippocampus," *Brain Res, vol.* 1256, pp. 111-122, 2009.
50. L.H. Tu, J. Ma, H.P. Liu, R.R. Wang, and J. Luo. "The neuroprotective effects of ginsenosides on calcineurin activity and tau phosphorylation in SY5Y cells," *Cell Mol Neurobiol, vol.* 29, no. 8, pp. 1257-1264, 2009.
51. Y.H. Qian, H. Han, X.D. Hu, and L.L. Shi. "Protective effect of ginsenoside Rb1 on beta-amyloid protein(1-42)-induced neurotoxicity in cortical neurons," *Neurol Res, vol.* 31, no. 7, pp. 663-667, 2009.
52. X. Xie, H.T. Wang, C.L. Li, et al. "Ginsenoside Rb1 protects PC12 cells against beta-amyloid-induced cell injury," *Mol Med Rep, vol.* 3, no. 4, pp. 635-639, 2010.
53. X. Chen, T. Huang, J. Zhang, J. Song, L. Chen, and Y. Zhu. "Involvement of calpain and p25 of CDK5 pathway in ginsenoside Rb1's attenuation of beta-amyloid peptide25-35-induced tau hyperphosphorylation in cortical neurons," *Brain Res, vol.* 1200, pp. 99-106, 2008.
54. Y. Wang, J. Liu, Z. Zhang, P. Bi, Z. Qi, and C. Zhang. "Anti-neuroinflammation effect of ginsenoside Rbl in a rat model of Alzheimer disease," *Neurosci Lett, vol.* 487, no. 1, pp. 70-72, 2011.
55. C.B. Wei, J.P. Jia, P. Liang, and Y.Q. Guan. "[Ginsenoside-Rg1 inhibits cell apoptosis induced by beta amyloid]," *Zhonghua Yi Xue Za Zhi, vol.* 88, no. 25, pp. 1763-1766, 2008.
56. Q. Liu, J.P. Kou, and B.Y. Yu. "Ginsenoside Rg1 protects against hydrogen peroxide-induced cell death in PC12 cells via inhibiting NF-kappaB activation," *Neurochem Int, vol.* 58, no. 1, pp. 119-125, 2011.
57. Y.Q. Shi, T.W. Huang, L.M. Chen, et al. "Ginsenoside Rg1 attenuates amyloid-beta content, regulates PKA/CREB activity, and improves cognitive performance in SAMP8 mice," *J Alzheimers Dis, vol.* 19, no. 3, pp. 977-989, 2010.

58. F. Chen, E.A. Eckman, and C.B. Eckman. "Reductions in levels of the Alzheimer's amyloid beta peptide after oral administration of ginsenosides," *FASEB J, vol.* 20, no. 8, pp. 1269-1271, 2006.
59. Y.H. Wang and G.H. Du. "Ginsenoside Rg1 inhibits beta-secretase activity in vitro and protects against Abeta-induced cytotoxicity in PC12 cells," *J Asian Nat Prod Res, vol.* 11, no. 7, pp. 604-612, 2009.
60. L. Yang, J. Hao, J. Zhang, et al. "Ginsenoside Rg3 promotes beta-amyloid peptide degradation by enhancing gene expression of neprilysin," *J Pharm Pharmacol, vol.* 61, no. 3, pp. 375-380, 2009.
61. S.H. Hwang, E.J. Shin, T.J. Shin, et al. "Gintonin, a ginseng-derived lysophosphatidic acid receptor ligand, attenuates Alzheimer's disease-related neuropathies: involvement of non-amyloidogenic processing," *J Alzheimers Dis, vol.* 31, no. 1, pp. 207-223, 2012.
62. J.H. Jang and Y.J. Surh. "Protective effect of resveratrol on beta-amyloid-induced oxidative PC12 cell death," *Free Radic Biol Med, vol.* 34, no. 8, pp. 1100-1110, 2003.
